# Assessment of level of knowledge and satisfaction of website about cleft lip and palate

**DOI:** 10.1590/2317-1782/e20240006en

**Published:** 2025-01-27

**Authors:** Melissa Picinato-Pirola, Raíssa Gomes Magalhães, Marilia Gabriela Gonçalves Ribeiro, Thiago Pestillo Seles, Camila de Castro Corrêa

**Affiliations:** 1 Faculdade de Ciências e Tecnologias em Saúde, Universidade de Brasília – UnB - Brasília (DF), Brasil.; 2 Faculdade de Arquitetura, Artes e Comunicação, Universidade Estadual Paulista – UNESP - Bauru (SP), Brasil.; 3 Universidade Vale do Rio Doce – UNIVALE - Governador Valadares (MG), Brasil.

**Keywords:** Computer Communication Networks, Health Promotion, Interdisciplinary Communication, Cleft Lip, Cleft Palate

## Abstract

**Purpose:**

To promote orientation about cleft lip and palate and to verify knowledge and satisfaction of an orientation program through a website developed for students and health professionals.

**Methods:**

This is a cross-sectional study, 13 healthcare professionals and 81 students from the areas of nursing, speech-language pathologist, medicine, nutrition, dentistry, and psychology participated. The research consisted of three stages: filling out a pre-program questionnaire, accessing the website (http://fissuralabiopalatina.unb.br/) developed by the researchers and filling out a post-program questionnaire. For the statistical analyses McNemar's, Chi-square and Fisher's Exact Tests were used.

**Results:**

The majority of students were enrolled in the eighth semester of graduation or above, with little or no prior contact with the CLP. After accessing the website, there was an increase in knowledge for eight questions (p≤0.05). 70.2% consider their satisfaction when accessing the website to be excellent, 24.5% very good and 5.3% good. 44.7% of participants praised the website's design and layout, accessible language and informative content.

**Conclusion:**

It was possible to promote the guidelines about cleft lip and palate on the website, observing was an expansion of the topic for students and health professionals.

## INTRODUCTION

Cleft lip and palate (CLP) is among the most prevalent craniofacial malformations^(1)^ and occurs due to the absence of fusion between the embryonic facial processes between the fourth and twelfth week of pregnancy^(2)^. Multifactorial etiology is involved, influenced by genetic and environmental factors, including genes associated with craniofacial formation and nutritional aspects, maternal stress, use of medications, absence or insufficient amount of folic acid and multivitamins, alcoholism and smoking during the embryonic period^(1,3)^.

This impairment stands out due to the complexity of it is aesthetic and functional effects in patients of cleft lip and palate. There are aspects that affect appearance, hearing and orofacial functions such as chewing, swallowing and speaking and can have a long-term influence on the quality of life of the patients who will require longitudinal care so that social integration can be achieved^(4)^.

An inter/multidisciplinary team with integrated methods is needed, in order to achieve the best prognosis and work with social inclusion throughout the process. Professionals such as physicians, dentists, nutritionists, speech-language pathologist (SLP), psychologists and nurses should be part of this team in order to offer adequate treatment, as well as the necessary guidance for caregivers and patients with CLP. In order to achieve this goal, it is essential that health professionals have adequate training to deal with patients suffering from craniofacial anomalies^(5)^.

Transformative actions in health education depend on the health care professional recognizing the importance of promoting and communicating information, as well as routine clinical practices for consolidating actions. These professionals have the undertaking of transforming their job with a generalist and humanitarian vision, being the communicators of information that provide physical, mental and social well-being and autonomy for sufferers^(6)^.

The health care area has taken advantage of the emergence and improvements in telecommunications technologies offering online services related to health, one of these being tele education, *i.e.* an educational model that uses information and communication technology (ICT) remotely to encourage and promote health care education^(7,8)^.

Among the educational resources provided are websites, websites allow ease of access, high level information flexibility by utilizing resources such as images, text, and videos that serve different purposes and offer the possibility of creating a virtual learning environment^(9,10)^.

There are not many publications about telehealth and CLP, especially within the area of SLP^(11)^. Consequently, there is a need to promote training initiatives for health professionals in order to improve management of patients with CLP and their caregivers.

The hypothesis of the present study is that through the availability of information about cleft lip and palate, there is an increase in the knowledge of students and professionals, in addition, that this form of presentation of knowledge can bring satisfaction in its access, contributing to motivation in training professional.

The purpose of this study is to promote orientation about cleft lip and palate and to verify knowledge and satisfaction of an orientation program through a website developed for students and health professionals.

## METHODS

This is a cross-sectional study that was approved by the Research Ethics Committee of the Faculdade de Ciências e Tecnologias em Saúde, Universidade de Brasília (UnB), CAAE: 02639718.3.0000.8093, number 3.159.051. All subjects participated voluntarily and signed the Free and Informed Consent Form.

The inclusion criterion stipulated that students had to be enrolled in courses in medicine, nursing, dentistry, SLP, nutrition or psychology, and be attending at least the 5th semester of the course at the UnB. These courses were selected because they are the most likely to be directly involved in hospital care for patients with cleft lip and palate. Healthcare professionals had to be linked to the University Hospital of Brasilia (HUB), being hired, volunteers, residents or university professors. Those who did not meet the inclusion criteria and who did not participate in all the stages of the study were excluded.

The sample consisted of 81 students, 15 men and 66 women with an average age of 22.4 years, from the following areas, (10) medicine, (11) nursing, (12) dentistry, (28) SLP, (11) nutrition and (9) psychology. In addition to 13 healthcare professionals, all women with an average age of 41.2 years from the following areas, (2) medicine, (5) nursing, (2) dentistry, (2) SLP and (2) nutrition.

Participants’ data were obtained through online questionnaires (students) and in person (health professionals), before and after accessing the website. The research was carried out online and consisted of three stages: the completion of a questionnaire before the information program Fissura Online, access to the website, and the completion of the questionnaire after the information program. Considering that the questionnaires would be completed online, and access to the website would also be unsupervised by the researchers, a script guiding the research procedures was sent, guiding the importance of carrying out each step with attention and care.

### Completion of the Fissura Online pre-program questionnaire

For the recruitment of students, the pre-program orientation questionnaire Fissura Online was developed using Google Forms and was distributed on social networks, especially in groups and media used by students at UnB. Initially data collection from health professionals was also intended to be done virtually, but due to a poor take up the data collection took place in person at HUB by a single researcher.

During recruitment of the students and health care professionals a form was made available with sociodemographic questions such as cell phone number with WhatsApp contact, academic background, work in the health area and 16 questions about the participants knowledge of cleft lip and palate. The questionnaire used in this study was developed in a previous study^(10)^, *i.e*., this form underwent an evaluation process by specialized professionals with clinical practice in the area of cleft lip and palate and after that any necessary adjustments were made (Table 1)^(10)^.

**Table 1:** Pre-program and post-program Fissura Online questionnaire

Q1. In your opinion, what is isolated cleft lip and palate:
( ) Disability ( ) Syndrome ( ) Malformation ( ) don't know
Q2. Do you consider that the incidence of cleft lip and palate is:
( ) Rare (1:650,000 live births) ( ) Common (1:650 live births) ( ) don't know
Q3. Is the cause of cleft lip and palate known?
( ) Yes ( ) No
Q3.1.Specify what you believe to be the cause of cleft lip and palate:
Q4. Cleft lip and palate occurs at which stage of the gestational period:
( ) Up to 12 weeks ( ) From 12 weeks to 21 weeks ( ) From 26 to 30 weeks ( ) From 34 weeks ( ) don't know
Q5. By what name do you know the cleft lip and palate?
( ) Cleft lip and palate ( ) Cleft lip ( ) Hair lip ( ) don't know
Q6.Can the diagnosis be performed prenatally?
( ) Yes ( ) No ( ) don't know
Q7. What is the best way to diagnose cleft palate after birth?
( ) Two-dimensional ultrasound ( ) Three-dimensional ultrasound ( ) Radiography ( ) Cephalometry ( ) Magnetic resonance imaging ( ) Tomography ( ) Clinical/intraoral evaluation ( ) don't know
Q8. Are there any restrictions on the type of delivery for children with isolated cleft lip and palate?
( ) Yes ( ) No
Q9. Do you believe that the treatment of patients with cleft lip and palate depends on a multidisciplinary team?
( ) Yes ( ) No ( ) don't know
Q9.1. Which professionals do you believe are involved in cleft treatment?
Q10. For the correction of cleft lip and palate, surgical procedures called primary surgeries are performed, which usually occur:
( ) In the first days of life ( ) Up to 6 months ( ) Up to the first year of life ( ) don't know
Q11. Can babies with a cleft lip be breastfed?
( ) Yes ( ) No ( ) don't know
Q12. Is the use of a tube at birth recommended for babies with cleft lip and palate without other impairments?
( ) Yes ( ) No ( ) don't know
Q13. Are sufferers with cleft palate more likely to develop hearing disorders?
( ) Yes ( ) No ( ) don't know
Q14. Do you think that the speech of children with cleft lip may be altered after surgery?
( ) Yes ( ) No
Q15. Do all children with cleft lip and palate need speech therapy for speech?
( ) Yes ( ) No ( ) don't know
Q16. Should oronasal hygiene be performed in children with cleft lip and palate?
( ) Yes ( ) No ( ) don't know
Q17. Did you consider the orientation program relevant?*
( ) Yes ( ) No ( ) don't know
Q18. How would you rate the Orientation Program on a scale of 0 to 5 (0 being poor and 5 being excellent)?*
( ) 1 ( ) 2 ( ) 3 ( ) 4 ( ) 5
Q19. Please leave your suggestion, criticism or praise of the orientation program here:*

(*)Questions asked only in the post-program questionnaire

**Caption:** Q- question

### Website access

After completing the pre-program questionnaire, the participants were contacted via WhatsApp (A cross-platform centralized instant messaging and voice-over-IP service), and given the website domain name: http://fissuralabiopalatina.unb.br/^(12)^ to access the website which was developed in a step prior to the application of this methodology. The detailed description of the website development was described in a previous study^(10)^. Students and health care professionals were given 3 days to access and study the website after which if they did not complete this step a researcher would contact them. If this step was still not completed after the researcher had contacted them three times within a period of two weeks they were excluded from the research.

When accessing the website, the research participants were able to see that the content is divided into 7 sections: purpose of the website, definition, causes, treatments, curiosities, types of clefts and FAQs (frequently asked questions), the last two being divided into subsections, which include cleft lip and palate, submucous cleft, eating, hearing and speech. In addition the website has a quick access glossary of terms used, definition of SLP, authors, bibliography and contact details^(10)^.

### Completion of the post-program questionnaire

At the end of the previous two steps, the Fissura Online information post-program questionnaire was sent out to the participants, which contained the same questions as the Fissura Online information pre-program questionnaire. This meant that a comparison could be made about the participants' level of knowledge before and after accessing the website (Table 2). Additionally questions were asked about participant satisfaction of the website and the website content.

**Table 2:** Sample Characteristics

Undergraduate Semester (Students)	SLP		Nursing		Dentistry		Psychology		Medicine		Nutrition	
	n	%	n	%	n	%	n	%	n	%	n	%
5th	7	25	1	9.1	2	17	4	45	0	0	2	18
6th	6	21	2	18.2	2	17	0	0	1	10	2	18
7th	7	25	1	9.1	2	17	1	11	1	10	2	18
8th	8	29	4	36.3	4	32	1	11	1	10	3	28
9th	0	0	1	9.1	2	17	1	11	4	40	2	18
10th	0	0	2	18.2	0	0	2	22	2	20	0	0
12th or greater	0	0	0	0	0	0	0	0	1	10	0	0
Total	28	100	11	100	8	100	9	100	10	100	11	100
**FLP content during graduation (Students)**													
Yes, just one class	1	3.6	0	0	0	0	0	0	1	10	1	9.1
Yes, some classes	15	53.6	5	45.4	9	75	0	0	4	40	0	0
Yes, in one semester	3	10.7	0	0	0	0	0	0	1	10	0	0
In more than one semester	9	32.1	0	0	2	16.7	0	0	0	0	0	0
was not given	0	0	3	27.3	1	8.3	9	100	2	20	9	81.8
I don’t remember	0	0	3	27.3	0	0	0	0	2	20	1	9.1
Total	28	100	11	100	12	100	9	100	9	100	11	100
**Link with HUB** * **(Professionals)**												
Public tender	2	100	2	40	1	50	0	0	0	0	2	100
Hired	0	0	0	0	0	0	0	0	0	0	0	0
Voluntary	0	0	3	60	1	50	0	0	0	0	0	0
Resident	0	0	0	0	0	0	0	0	2	100	0	0
College professor	0	0	0	0	0	0	0	0	0	0	0	0
Total	2	100	5	100	2	100	0	0	2	100	2	100
**Working time** * **(Professionals)**												
Less than 1 year	0	0	1	20	1	50	0	0	0	0	0	0
1 to 2 years	0	0	2	40	0	0	0	0	1	50	0	0
2 to 5 years	0	0	1	20	0	0	0	0	1	50	0	0
5 to 7 years	0	0	1	20	1	50	0	0	0	0	0	0
7 to 10 years	0	0	0	0	0	0	0	0	0	0	0	0
More than 10 years	2	100	0	0	0	0	0	0	0	0	2	100
Total	2	100	5	100	2	100	0	0	2	100	2	100

**Caption:** SLP Speech-language pathologist

* Questions for professionals only

### Data analysis

The statistical analysis of the data was computed in tables and the calculation of absolute (N) and relative (%) frequencies were used to describe the variables studied. When analyzing the associations between the variables, as well as comparing the effectiveness of the Fissura Online information program, the McNemar, Chi-Square and Fisher's Exact Test were used with Jamovi software, version 2.0.0^(13)^. All differences were considered statistically significant at a significance level of 5%. In the analysis of the discursive questions, it was necessary to group the answers. For this purpose the researcher evaluated the main message of each answer categorizing those with the same meaning.

## RESULTS

Most of the students in this research were enrolled in their eighth semester of graduation or above. In addition, the data collected showed that undergraduates had little or no contact with CLP, especially psychology students, who reported never having had classes on the subject. As for the 13 health care professionals in this research, four of them had 10 years or more work experience (Table 2).

After the guidance Fissura Online program and the statistical analysis of the absolute and relative frequencies (Table 3), it was found that there was a significant difference in eight questions, that is, half of the total number of questions applied (p≤0.05).

**Table 3:** Comparison of participants' answers in the pre-program and post-Fissura Online questionnaires

		Pre-program		Post-program		P-value
		N	%	N	%	
Q1#	Deficiency	0	0	1	1.1	0.739
	Syndrome	0	0	1	1.1	
	Malformation	92	97.8	92	97.8	
	Don’t know	2	2.2	0	0	
Q2*#	Rare	18	19.2	8	8.5	0.009
	Common	65	69.2	83	88.3	
	Don’t know	11	11.6	3	3.2	
Q3*	Yes	65	69.2	28	29.8	< .001
	No	29	30.8	66	70.2	
Q4#	Up to 12 weeks	54	57.5	84	89.3	0.176
	From 12 to 21 weeks	16	17	6	6.4	
	From 26 to 30 weeks	3	3.2	0	0	
	From 34 weeks	0	0	1	1.1	
	Don’t know	21	22.3	3	3.2	
Q6#	Yes	68	72.3	91	96.8	< .001
	No/Don't know	26	27.7	3	3.2	
Q8*	Yes	5	5.3	6	6.4	0.739
	No					
		89	94.7	88	93.6	
Q10+	In the first days of life	7	7.5	4	4.3	0.115
	Up to 6 months	23	24.5	16	7	
	Up to one year old	48	51	73	77.7	
	Don’t know	16	17	1	1	
Q11#	Yes	59	62.8	84	89.4	< .001
	No/Don't know	35	37.2	10	10.6	
Q12#	Yes	14	14.9	4	4.2	< .001
	No/Don't know					
		80	85.1	90	95.8	
Q13#	Yes	56	59.6	88	93.6	< .001
	No/Don't know	38	40.4	6	6.4	
Q14*	Yes	81	86.1	67	72.3	0.007
	No	13	13.9	27	28.7	
Q15*	Yes	60	63.8	44	46.8	0.012
	No	34	36.2	50	53.2	
Q16*	Yes	90	95.8	89	94.7	0.157
	No	4	4.2	5	5.3	

* McNemar test;

#- Chi-square test;

+: Fisher's exact test

**Caption:** Q- question, N- absolute value, %, relative value

**Figure 1:**
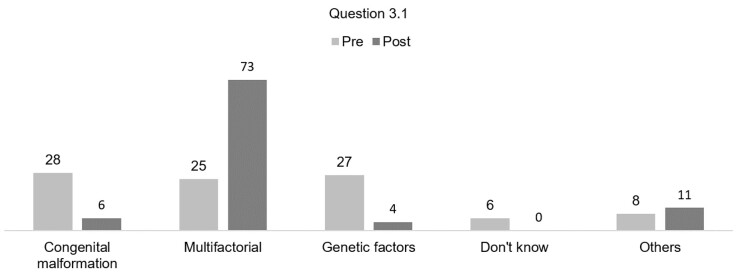
Knowledge of students and health professionals about the etiology of cleft lip and palate

Regarding the etiology of cleft lip and palate (Q3), there was an increase in answers related to the multifactorial cause after the Fissura Online program. In the “others” category, as shown in Figure 1, the participants mentioned factors such as teratogenic factors, nutrition during pregnancy, consanguinity and maternal age.

**Figure 2:**
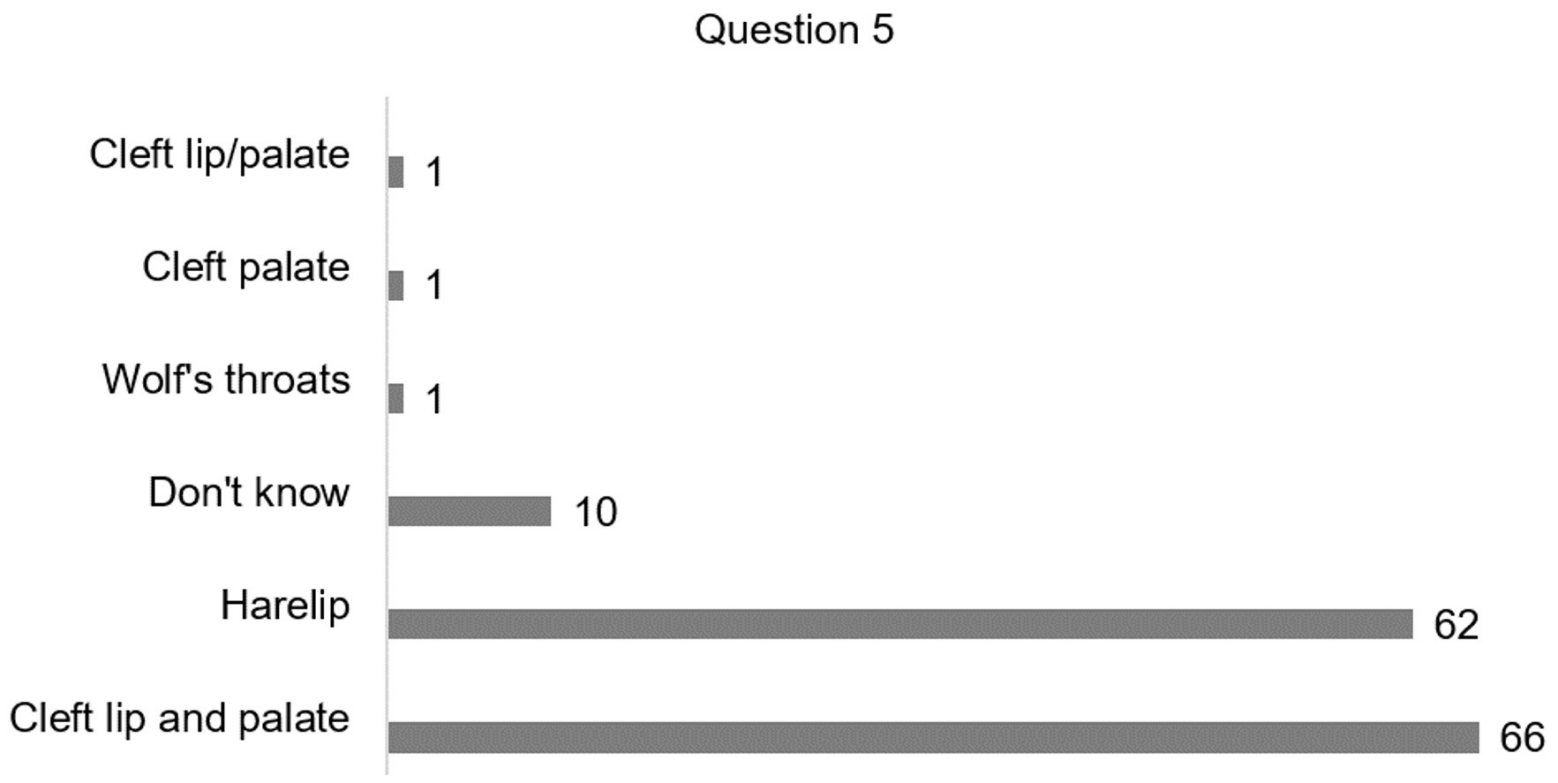
Knowledge of students and health professionals about the nomenclature of cleft lip and palate

Regarding prior knowledge about the nomenclature of the malformation (Q5 - Figure 2), most participants reported that they knew it as cleft lip and palate, followed by harelip. Another 10 participants marked “I don't know”, indicating that they did not know the correct nomenclature, or possibly know anything about CLP.

**Figure 3:**
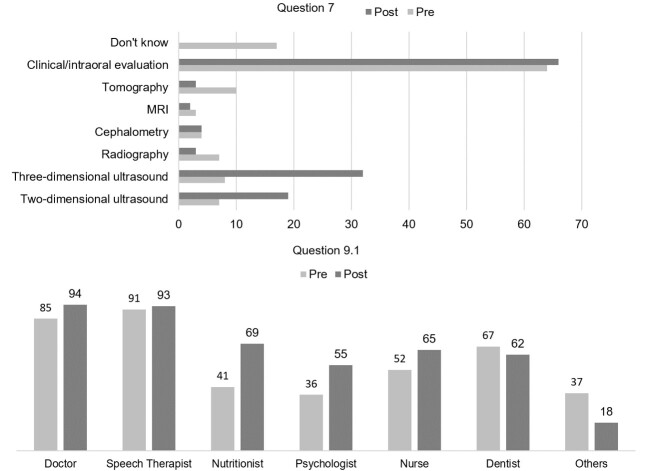
Question 7 there was comparison between pre and post-Fissura Online program answers regarding the diagnosis of cleft lip and palate after birth. Question 9.1 there was a comparison between pre- and post-Fissura Online responses in relation to professionals involved in the treatment of cleft lip and palate

When asked about the diagnosis of CLP after birth (Q6 - Figure 3), there was a higher number of responses related to the clinical/intraoral assessment that was found in both questionnaires, there was an increase after the guidance Fissura Online program. There were an even greater number of responses citing two-dimensional and three-dimensional ultrasound in the post-program questionnaire (Q7). Added to this, there were few “I don't know” answers after the program.

Regarding the multidisciplinary team involved in the treatment of patients with CLP (Q9 - Figure 3), the physicians most mentioned were specialists such as otorhinolaryngologist, pediatrician, geneticist and plastic surgeon. SLP were also mentioned by 98.9% of the participants after the program, compared to 96.8% before the program. The analysis showed a 40% increase in the nutritionists being cited in the treatment of individuals with CLP. Other professionals were also mentioned such as occupational therapists, physical therapists, pedagogues and social workers, who were classified as “others”.

As for the questions regarding participants' satisfaction about the guidance Fissura Online program (Q17 to Q19), all of the participants rated the program, with 70.2% considering it excellent, 24.5% very good and 5, 3% good. The last question (Q19), the participants were able to express their opinions about the program, and 44.7% of the participants praised the design and layout, accessible language and informative content of the website. Another 13 participants pointed out difficulties in accessing the website content from smartphones, and the use of technical terms in some sections of the website. In addition, there were 8 suggestions that cited the inclusion of actual photos and videos of the CLP classification, and the assessment of occult submucosal cleft.

## DISCUSSION

The Fissura Online orientation program developed during this research aimed to disseminate information to students and health care professionals to promote health in a broader perspective. Not only focusing on the prevention of diseases and injuries, but also on aspects of quality of life^(13)^. To achieve this aim technological resources based on information and communication technologies^(7,8)^ were used, through tele-education with the development of the cleft lip and palate website^(10)^.

When considering the results of the research, it is was found that in general there was an increase in the frequency of correct answers after the Fissura Online program representing that the guidance program was positive in this application. In another study similar to this one^(14)^, results showed the effectiveness of a SLP orientation program targeting teachers about the subject of mouth breathing.

Table 2 shows that most of the students were on more advanced semesters of their undergraduate course, however at the time of this research, they had not had any orientation or classes about CLP. Among the professionals, there was a lack of information about CLP when checking the Fissura Online pre-program questionnaire. It is worth noting that treatment of this malformation requires the support of a multidisciplinary team comprised of all areas of knowledge involved in this research^(1)^, so the absence or insufficiency of knowledge about CLP can directly reflect on the quality of services provided to patients.

About the incidence of CLP (Q2), there is a significant increase in responses marked as “common”, with a significant difference, which shows an improvement in the level of knowledge by the participants after the Fissura Online program, considering that the incidence of this malformation is 1.65 per thousand births in Brazil^(1,15)^.

When asked about the cause of CLP (Q3), 69.2% believed that the cause was known, however after viewing the website, this number decreased to 29.8%, showing a significant difference when comparing the responses between the questionnaires. According to what is known the etiology of CLP is influenced by several factors such as alcohol abuse, smoking, folic acid deficiency and genetic factors^(3)^. Vyas et al.^(1)^, also emphasizes that CLP associated with syndromes are more closely related to genetic factors. In response Q3.1 after the Fissura Online program the participants cited that there was a higher frequency of multifactorial causes which corroborated existing information of what is known at the moment about the etiology of CLP^(1,16)^.

When the participants were asked about the period of occurrence of CLP (Q4), 57.5% marked the alternative “up to 12 weeks” before the Fissura Online program, and after the program there was an increase in the alternative “up to 12 weeks” to 89.3%, which corroborates what is already known^(15,17)^, consequently the Fissura Online program increased the participants' area of knowledge of the subject.

Regarding the nomenclature (Q5) used for the most common craniofacial malformation 46.8% of the participants said that they knew it as “cleft lip and palate”, followed by 44% who know it as “harelip”. The terminology "harelip" is an old term and more commonly used by lay people, and it refers only to the cleft of the lip. On the other hand, the term “cleft lip and palate” has been most widely used in clinical practice and in scientific research and this description covers both the failure of the palate and the upper lip^(1,18)^.

CLP diagnosis during pregnancy can be made by means of two-dimensional and three-dimensional ultrasound performed during prenatal care. Early diagnosis makes it possible to outline guidance and treatment strategies with the multidisciplinary team even before birth, with the aim of providing a better prognosis^(19)^. This subject was touched upon during the Fissura Online program and the study participants were questioned about this (Q6). In the Fissura Online pre-program questionnaire only 72.3% of the participants agreed with this information, and after the program this number increased to 96.8% of the participants in agreement with the diagnosis during prenatal care. Comparison of the answers show a significant difference.

When a diagnosis is not carried out during pregnancy due to difficulties in visualizing the cleft through the aforementioned exams, it can be performed after birth during the clinical and intraoral evaluation which is considered the most appropriate for this purpose^(20)^. Participants had access to this information on the website, and were also questioned in the pre and post-program Fissura Online questionnaires about postnatal diagnosis in (Q7), which showed that the participants already knew something about this topic in the pre-program questionnaire, added to an increase of only 2 correct answers in the post questionnaire. In addition, the increased number of answers in the post-program questionnaire related to diagnostic procedures used in prenatal care is highlighted, demonstrating the participants' confusion about the diagnostic methods performed during pregnancy and after birth.

There are no known restrictions for giving birth to babies with CLP and delivery can be performed normal or by cesarean delivery^(21)^. When participants were asked about this topic (Q8) the participants showed that they already knew something about it and for the most part, marked the alternative “no” in both questionnaires.

There were two questions (Q9 and Q9.1) about the treatment of CLP, which allowed the participants to show how much they already knew about this subject. Q9 showed how much the participants already knew by highlighting the unanimous and assertive response that the treatment involves a multidisciplinary team. This statement corroborates current findings that point to the need for a multi/disciplinary approach to CLP cases by a team composed of professionals such as physicians and dentists and from different specialties such as nursing, SLP, nutrition and psychology^(22)^.

Question (Q9.1) asked the participants to describe which professionals should form part of a multidisciplinary team. In the pre and post-Fissura Online questionnaires, greater mention was made of medical professionals and SLP, who play an important roles in lip and palate surgeries, in addition to the rehabilitation of orofacial functions that may be altered, such as speech, swallowing and chewing which require longitudinal monitoring^(1,23)^. However the team must rely on other professionals, such as nutritionists who plays a fundamental role in the nutritional aspects of sufferers especially during periods of surgery. When ascertaining what the students and health care professionals knew about the team involved in treatment, there was a significant increase in the mention of nutritionists in the post-program questionnaire compared to the other professionals^(24)^.

Still on the treatment of CLP and surgical correction of the lips (cheiloplasty) and palate (palatoplasty). Some studies^(4,25)^ show that cheiloplasty should be performed between 3 and 6 months of age, and palatoplasty between 9 and 12 months of age although there are differences of opinion about the correct age for carrying out surgery and differences among the reference center that perform CLP surgery, it is generally agreed that both surgeries must be performed within the first year of life. Therefore participants in the study were asked in both questionnaires (Q10) to state how much they knew about the subject. It was noted that the alternatives "up to 6 months of life" and "up to one year of life" were the most popular answers in the two questionnaires, and after the Fissura Online program there was an increase of 26.7% who considered that primary surgery is usually carried out during the first year of life.

The degree of difficulty in breastfeeding babies with CLP (Q11) may well depend on the type of cleft and the structures affected, babies affected with cleft lip tend to have less difficulty than those with cleft palate, this is due to the maintenance of intraoral pressure necessary for efficient suction^(1)^. Question 11 (Q11) addresses dietary aspects, showing a marked decrease in “No/I don't know” answers in the post-program questionnaire, highlighting the significant difference pointed out after Fissura Online program.

Considering aspects of food and nutrition Q12 asks about the use of Gavage feeding by tube of babies with CLP. It should be noted that the requirement for Gavage feeding depends on the multidisciplinary team and takes into consideration both physiological and nutritional aspects. The use of tubes such as Nasogastric tubes (NG Tube) can cause respiratory, suction and swallowing changes therefore their use should be restricted when possible or even used only for the shortest time necessary. Therefore children with CLP do not need to be Gavage fed unless they have feeding difficulties, syndromes and/or neurological alterations that can justify the use of a tube^(24)^. The Fissura Online program proved to be very effective in disseminating information about the use of the probe and this was seen by the decrease in “yes” answers in the pre-program Fissura Online questionnaire and by the significant difference pointed out in the statistical analysis.

Sufferers of cleft palate are more likely to develop hearing disorders due to inadequate insertion of the tensor veli palatini and levator veli palatini muscle in the palate, which can cause tubal dysfunction, in other words failure of the Eustachian tube mechanism, consequently probability of otitis media and other hearing disorders increases significantly, impacting the development of speech and language^(26,27)^. When participants were asked about the subject of cleft palate sufferers being more prone to hearing disorders, 59.6% of the participating students and health care professionals agreed with the information before the Fissura Online program, increasing to 93.6% after the program, showing a significant difference when comparing the answers between the questionnaires.

It is known that cleft lip does not cause changes in speech, however, cleft palate can cause velopharyngeal dysfunction (VPD) which modifies speech by developing mandatory disorders, such as: hypernasality, weak intraoral pressure and nasal air leakage^(28)^, therefore these sufferers require SLP to minimize the changes that directly interfere with speech. Most of the participants in this study marked “yes”, in agreement that patients with cleft lip will have speech changes after cheiloplasty. This fact disagrees with what is known since cleft lip after cheiloplasty does not have consequences of speech impairment^(29)^. In Q15, the participants showed an increase in their knowledge after the program proved by the quantitative increase in the “no” answer in the questionnaire after accessing the Fissura Online program.

Oronasal hygiene was also an issue addressed in the study, in Q16 the participants were asked about its effect on CLP patients and they marked the alternative “yes” in both questionnaires. This statement is supported by current findings, emphasizing that oronasal hygiene is necessary for CLP sufferers and gives benefits such as desensitization in the cleft lip and palate region and aids the healing process after primary surgery. In addition, it is important to perform nasal cleaning due to the nasal reflux that occurs before performing primary surgery which helps to avoid the possibility of post surgery infections^(30)^.

Finally questions (Q17, Q18 and Q19) of the Fissura Online post-program questionnaire about satisfaction showed that the majority of participants were satisfied with the program, and proved an absence of this content during graduation, given that described in Table 2, which emphasizes the need for greater dissemination of information about CLP, especially for undergraduates in the health area who, in their professional activities will be able form part of a multidisciplinary team involved in the treatment of this malformation.

There are very few studies similar to this one whose purpose is to inform about CLP, even more so using technologies such as tele-education and websites. Therefore, it is important to highlight the relevance of this research, as it contributed significantly to the knowledge of professionals and students in the health area, when comparing the pre and post-Fissura Online questionnaires, which shows the relevance of health care promotion.

It should be noted that the present study had the limitation of its sample being from a single location, in addition to low control when filling out the questions and accessing the educational material. Therefore, for future studies, it is suggested to randomize the sample, considering different geographic locations, as well as completing and accessing the material with synchronous monitoring, even if remotely. It should also be noted that the long-term effectiveness of knowledge after accessing the website was not evaluated.

## CONCLUSION

Considering the multidisciplinary team in the treatment of cleft lip and palate, knowledge about CLP was disseminated to undergraduate students and professionals, in which this educational tool showed positive results to half of the questions, as well as satisfaction in its use.
